# Risk Factors Associated with Incident Syphilis in a Cohort of High-Risk Men in Peru

**DOI:** 10.1371/journal.pone.0162156

**Published:** 2016-09-07

**Authors:** Hayoung Park, Kelika A. Konda, Chelsea P. Roberts, Jorge L. Maguiña, Segundo R. Leon, Jesse L. Clark, Thomas J. Coates, Carlos F. Caceres, Jeffrey D. Klausner

**Affiliations:** 1 UCLA David Geffen School of Medicine Program in Global Health, Los Angeles, CA, United States of America; 2 Universidad Peruana Cayetano Heredia, Unidad de Salud, Sexualidad y Desarrollo Humano, Lima, Peru; 3 Escuela de Medicina, Universidad Peruana de Ciencias Aplicadas, Lima, Peru; Azienda Ospedaliera Universitaria di Perugia, ITALY

## Abstract

**Background:**

Syphilis is concentrated among high-risk groups, but the epidemiology of syphilis reinfection is poorly understood. We characterized factors associated with syphilis incidence, including reinfection, in a high-risk cohort in Peru.

**Methods:**

Participants in the NIMH CPOL trial were assessed at baseline and 2 annual visits with HIV/STI testing and behavioral surveys. Participants diagnosed with syphilis also attended 4- and 9-month visits. All participants underwent syphilis testing with RPR screening and TPPA confirmation. Antibiotic treatment was provided according to CDC guidelines. Reinfection was defined as a 4-fold titer increase or recurrence of seroreactivity after successful treatment with subsequent negative RPR titers. The longitudinal analysis used a Possion generalized estimating equations model with backward selection of variables in the final model (criteria P <0.02).

**Results:**

Of 2,709 participants, 191 (7.05%) were RPR-reactive (median 1:8, range 1:1–1:1024) with TPPA confirmation. There were 119 total cases of incident syphilis, which included both reinfection and first-time incident cases. In the bivariate analysis, the oldest 2 quartiles of age (incidence ratio (IR) 3.84; P <0.001 and IR 8.15; P <0.001) and being MSM/TW (IR 6.48; P <0.001) were associated with higher risk of incident syphilis infection. Of the sexual risk behaviors, older age of sexual debut (IR 12.53; P <0.001), not being in a stable partnership (IR 1.56, P = 0.035), higher number of sex partners (IR 3.01; P <0.001), unprotected sex in the past 3 months (IR 0.56; P = 0.003), HIV infection at baseline (IR 3.98; P <0.001) and incident HIV infection during the study period (IR 6.26; P = 0.003) were all associated with incident syphilis. In the multivariable analysis, older age group (adjusted incidence ratio (aIR) 6.18; P <0.001), men reporting having sex with a man (aIR 4.63; P <0.001), and incident HIV infection (aIR 4.48; P = 0.008) were significantly associated.

**Conclusions:**

We report a high rate of syphilis reinfection among high-risk men who have evidence of previous syphilis infection. Our findings highlight the close relationship between HIV incidence with both incident syphilis and syphilis reinfection. Further studies on syphilis reinfection are needed to understand patterns of syphilis reinfection and new strategies beyond periodic testing of high-risk individuals based on HIV status are needed.

## Introduction

HIV and syphilis co-infection is a significant public health problem. In previous public health surveillance data, HIV-positive individuals had co-infection rates of 18.9% with positive TPPA testing and 5.3% with recent syphilis (defined as RPR ≥ 1:8) in Brazil [1]. In Peru, Lama *et al*. found a strong association between past syphilis diagnosis and HIV infection among men who have sex with men (MSM) [2]. The iPrex trial also showed a significant association between syphilis incidence and HIV incidence [3]. Pathela *et al*. showed increased risk of HIV diagnosis after primary or secondary syphilis diagnosis in New York [4]. Multiple epidemiologic studies have demonstrated an association between syphilis infection and risk of HIV transmission and acquisition, highlighting the link between these two infections [5–7].

In Peru, syphilis is concentrated among high-risk groups, including MSM and male-to-female transgender women (TW), similar to the country’s HIV epidemiology. A 2005 study of MSM and TW in Lima, Peru reported recent syphilis (RPR titer ≥ 1:8) prevalence at 10.5% [8]. In contrast, a population-based survey of Peruvian young adults in 2002 reported the prevalence of recent syphilis (RPR titer ≥ 1:8) at 0.5% and in 2004, lifetime history of syphilis prevalence was 1.5% (any RPR titer) among a sample of male partners of pregnant women [9, 10].

Most previous studies of syphilis reinfection and incidence have been limited to cross-sectional analyses. A retrospective study of all the cases of syphilis in San Francisco among MSM reported a 6.7% rate of reinfection between 2001 and 2002 and found that HIV prevalence was associated with repeat episodes of syphilis infection after adjustment for age and ethnicity [11]. In British Columbia, a group reported a syphilis reinfection incidence rate of 2.0 per 100 person years over 10 years of STI surveillance with risk for repeat infection associated with HIV status, MSM behavior, and history of other sexually transmitted infections [12].

We conducted a prospective longitudinal analysis of high-risk men in Peru to determine the incidence of syphilis and the associated high-risk behaviors and other STDs. Our analysis has expanded the frame of a previous analysis to include two years of follow-up data estimating the incidence of syphilis infection, including reinfection [13].

## Methods

### Study design and population

We conducted a longitudinal study of syphilis infection and reinfection among participants in the Peru site of the National Institute of Mental Health (NIMH) Collaborative HIV/STD Prevention Trial [14–16]. Briefly, participants of ethnographically-identified sub-populations at high risk for HIV acquisition were recruited from low-income barrios in three urban, coastal cities: Lima, Trujillo and Chiclayo, Peru between 2002 and 2005. Those subpopulations were: “*Esquineros*” or “Corner Men”, non-gay-identified, socially-marginalized males who often engaged in activities such as petty theft, drug use and sales, and compensated sex with male or transgender partners, which included gay-identified men who have sex with men (MSM) and/or male-to-female transgender women (TW). The protocol used in this study was approved by the Committee of Human Research of University of California, Los Angeles; and Cayetano Heredia University, Lima, Peru. Written informed consent was obtained and filed separate from the participant’s study data. The data is available under the identifier NCT00810060 (https://clinicaltrials.gov/ct2/show/NCT00710060)) and was presented at the 2015 American Public Health Association annual conference.

HIV and STI testing and behavioral surveys were conducted at Baseline, 12- and 24-month follow-up visits. Behavioral surveys were conducted at each visit by trained study staff using Computer Assisted Personal Interviewing (CAPI) in private and in Spanish. Survey questions assessed socio-demographic information, details of alcohol and drug use history, and sexual risk behaviors with the last five sex partners.

### Laboratory methods and clinical management

Blood, vaginal swabs and urine samples were obtained from participants at each annual study visit and transported to the US Naval Medical Research Center Detachment in Lima, Peru for testing. Lab personnel followed manufacturers’ specifications and protocols. Syphilis testing was done using RPR-nosticon II Rapid Plasma Reagin kits (Biomerieux, Boxtel, Netherlands) with *Treponema pallidum* Particle Agglutination assay confirmation using Serodia-TPPA (Fujirebio Diagnostic Inc, Toyko, Japan) and RPR titer determined by serial dilutions. HIV testing was conducted using Genetic Systems HIV-1/HIV-2 Peptide EIA (BioRad, Hercules, CA) with Western blot confirmation (Genetic Systems; BioRad) of positive specimens.

Participants diagnosed with syphilis based on serology were given weekly injections of benzathine penicillin G 2.4 million units IM (once for primary or secondary infection and three times for late latent infection) or doxycycline 100 mg PO twice daily for two to four weeks, if unable to tolerate penicillin. Participants diagnosed with syphilis were also asked to attend additional interim visits at four and nine months after treatment to conduct repeat serology testing and to assess persistent infection or reinfection. Participants found to have treatment failure or reinfection at any of these visits (using the criteria described below) were provided with an additional course of antibiotic therapy. No additional behavioral or biological data were collected at these interim visits.

### Variables used

We analyzed biological and behavioral data collected at baseline and annual follow-up visits. In the descriptive analysis, participants were re-categorized into sub-groups according to their recent self-reported sexual behavior: men who reported sex with only women (MSOW) and men who reported sex only with men and/or transgender women (MSM/TW) in order to better reflect the association of HIV and STI risk with sexual behavior [17].

Descriptive variables included limited access to food, which was re-categorized as “Yes” (“rarely” or “never” experience of food instability) and “No” (“at least once a month”/“at least once a week”/“everyday” experience of food instability). Work stability was categorized as stable work as “Yes” and occasional work or financial support from others as “No”.

Behavioral data assessed sexual risk behaviors during the previous three months with up to five sex partners. Number of sexual active years was calculated from age of sexual debut and age at baseline. The total number of sex partners in the last six months was calculated including stable and non-stable partnerships. Stable partnership was defined if sex partners were identified as a spouse or live in partner and unstable partnership as those with who were not. Assessment of alcohol and drug use was based on self-reported behavior before sex in the last 10 sex acts with up to five partners.

Incident syphilis infection was defined as any new RPR/TPPA-positive result at the 12 or 24-month follow-up visit following a previous negative RPR titer result. Syphilis reinfection was defined as either: a) a four-fold increase in RPR titer or b) a positive RPR test following successful antibiotic treatment that was confirmed by a four-fold decline in RPR titer or loss of RPR seroreactivity. Probable reinfection was defined as a small titer increase (1:1–1:2) after nonreactive RPR titer status post treatment. First-time incident infection was defined as any RPR seroreactivity at the 12 or 24-month follow-up visits following negative results during all previous visits.

### Data analysis

Descriptive analysis of groups were stratified according to syphilis status and associated demographic and sexual behaviors, which were based on the “life-time” report of all of the follow-up data, unless specified. Repeat syphilis incidence rate per 100 person-years (PY) was calculated for the participants with documented history of syphilis infection either as baseline or during a study visit. The syphilis incidence rate per 100 PY was calculated for the participants who attended at least one follow-up visit.

The longitudinal analyses used a Poisson generalized estimating equations model with an exchangeable correlation structure. For the incident syphilis outcome, the categorical variable for herpes simplex virus (HSV) prevalence and incidence were not included as their colinearity with HIV infection interfered with model convergence. Variables with a p-value of ≤0.20 were included in the multivariable model. To reach the most parsimonious model, non-significant variables were then removed until all variables in the model were significantly associated with the outcome of interest with a p-value of <0.05. Similar analyses were performed for the outcome of syphilis reinfection. To further elucidate the association of HIV incidence with incident syphilis, several models were explored controlling for different groups of potential confounders. All statistical analysis was conducted in Stata 13 (College Station, TX).

## Results

### Descriptive analysis

There were 2,709 participants with at least one follow-up visit; 2,587 (95.5%) participants came to follow-up visit 1 and 2,372 (87.6%) participants came to follow-up visit 2. There were 2,518 (92.9%) participants with no reactive titer at any visit. At baseline, 159 (5.9%) participants had reactive RPR titers; of those 72 (45.2%) participants did not have a reinfection during follow-up. There were a total of 89 (56.0%) participants with reinfection during the study period. Of these participants with reinfection, 25 (28%) participants had probable reinfection with a low rise in titers after confirmed nonreactive RPR titers status post treatment. There were 30 (1.1%) participants who had first-time incident syphilis during the study period. Prevalence of HIV infection at baseline was 2.8%. The syphilis incidence rate was 2.3 per 100 person years and syphilis reinfection rate was 35.3 per 100 person years. Description of the participants based on ever-reported characteristics during the study period are shown in Table 1.

**Table 1:** Ever reported characteristics based on syphilis infection among high-risk men in Peru.

Variables	No syphilis infection (n = 2518)	Reactive at baseline with no reinfection (n = 72)	First-time incident syphilis (n = 30)	Syphilis reinfection (n = 89)
Age at baseline				
Mean±SD	23.8±5.3	25.9±5.6	30.2±5.8	28.6±5.3
Participant type				
MSOW	1750 (69.5)	17 (23.6)	9 (30.0)	20 (22.5)
MSM	768 (30.5)	55 (76.4)	21 (70.0)	69 (77.5)
High school education at baseline				
No	1210 (48.1)	33 (45.8)	14 (46.7)	47 (52.8)
Yes	1308 (51.9)	39 (54.2)	16 (53.3)	42 (47.2)
Reporting having stable work at any visit				
No	1357 (53.9)	26 (36.1)	10 (33.3)	35 (39.3)
Yes	1161 (46.1)	46 (63.9)	20 (66.7)	54 (60.7)
Food instability at any visit				
No	1655 (65.7)	49 (68.1)	24 (80.0)	69 (77.5)
Yes	863 (34.3)	23 (31.9)	6 (20.0)	20 (22.5)
Age of sexual debut				
Mean±SD	15.2±2.7	13.8±2.7	14.3±2.8	14.0±3.0
Engaged in compensated sex, past 3 months of visit				
No	1036 (41.1)	53 (73.6)	20 (66.7)	62 (69.7)
Yes	396 (15.7)	19 (26.4)	10 (33.3)	24 (27.0)
In a stable partnership at any visit				
No	1124 (44.6)	29 (40.3)	12 (40.0)	38 (42.7)
Yes	1340 (53.2)	42 (58.3)	18 (60.0)	51 (57.3)
Avg. no. of sex partners at all visits, past 6 months of visit				
Mean±SD	3.2±13.8	10.3±33.1	28.3±76.4	10.7±24.1
Any unprotected sex acts past 3 months of visit				
No	476 (18.9)	22 (30.6)	9 (30.0)	24 (27.0)
Yes	1763 (70.0)	48 (66.7)	20 (66.7)	59 (66.3)
Substance use before sex in last 10 sexual encounters				
No	870 (34.6)	21 (29.2)	8 (26.7)	31 (34.8)
Yes	1577 (62.6)	51 (70.8)	22 (73.3)	58 (65.2)
HIV infection				
Negative	2430 (96.5)	59 (81.9)	25 (83.3)	78 (87.6)
Prevalent at baseline	53 (2.1)	12 (16.7)	5 (16.7)	6 (6.7)
Incident	15 (0.6)	1 (1.4)	0 (0.0)	5 (5.6)

### Longitudinal analysis of incident syphilis infection

There were 119 total cases of incident syphilis, which included both reinfection and first-time incident cases. In the bivariate analysis, the oldest 2 quartiles of age, which includes ages 24–27 and greater than 28 years of age (incidence ratio [IR] 3.84; *P* <0.001 and IR 8.15; *P* <0.001) and being an MSM/TW (IR 6.48; *P* <0.001) were significantly associated with higher risk of incident syphilis infection (see Table 2). Of the socio-demographic variables, not having stable work was associated with higher risk of incident syphilis (IR 1.67; *P* = 0.004). Of the sexual risk behaviors, older age of sexual debut (IR 12.53; *P* <0.001), not being in a stable partnership (IR 1.56, *P* = 0.035), higher number of sex partners (IR 3.01; *P* <0.001), and unprotected sex in the past 3 months (IR 0.56; *P* = 0.003) were all associated with incident syphilis. Both HIV infection at baseline (IR 3.98; *P* <0.001) and incident HIV infection during the study period (IR 6.26; *P* = 0.003) were significantly associated with increased risk of incident syphilis.

**Table 2:** Longitudinal analysis of associations with syphilis incidence among high-risk men in Peru.

Variable	Crude Cumulative Incidence Ratio (95% CI)	P value	Adjusted Cumulative Incidence Ratio (95% CI)	P value
Age				
18–20	*ref*		ref	
21–23	*0*.*96 (0*.*38–2*.*41)*	*0*.*935*	0.90 (0.35–2.31)	0.830
24–27	*3*.*84(1*.*90–7*.*79)*	*<0*.*001*	**3.16 (1.54–6.45)**	**0.002**
28+	*8*.*15 (4*.*18–15*.*89)*	*<0*.*001*	**6.18 (3.15–12.14)**	**<0.001**
Participant type				
MSOW	*ref*		ref	
MSM	*6*.*48 (4*.*22–9*.*93)*	*<0*.*001*	**4.63 (2.98–7.21)**	**<0.001**
High school education				
No	ref			
Yes	0.94 (0.65–1.36)	0.738		
Reporting having stable work*				
Yes	*ref*			
No	*1*.*67 (1*.*18–2*.*36)*	*0*.*004*		
Food stability*				
No	ref			
Yes	0.81 (0.55–1.18)	0.275		
Age of sexual debut in quartiles				
0–14	*ref*			
15	*1*.*97 (0*.*76–5*.*10)*	*0*.*166*		
16–17	*4*.*44 (1*.*96–10*.*07)*	*<0*.*001*		
18+	*12*.*53 (5*.*83–26*.*94)*	*<0*.*001*		
Engaged in compensated sex, past 3 months*				
No	*ref*			
Yes	*1*.*51 (0*.*99–2*.*33)*	*0*.*058*		
Stable partnership*				
Yes	*ref*			
No	*1*.*56 (1*.*03–2*.*37)*	*0*.*035*		
No. of sex partners in tertiles, past 6 months*				
0–1	*ref*			
2	*1*.*21 (0*.*73–2*.*03)*	*0*.*461*		
3+	*2*.*77 (1*.*87–4*.*11)*	*<0*.*001*		
Any unprotected sex acts past 3 months*				
No	*ref*			
Yes	*0*.*60 (0*.*40–0*.*89)*	*0*.*011*		
Substance use before sex in last 10 sexual encounters*				
No	ref			
Yes	1.24 (0.84–1.84)	0.275		
HIV infection				
Negative	*ref*		ref	
Prevalent at baseline	*3*.*77 (2*.*04–6*.*96)*	*<0*.*001*	1.35 (0.72–2.52)	0.343
Incident	*5*.*84 (1*.*74–19*.*61)*	*0*.*004*	**4.48 (1.47–13.59)**	**0.008**

*Time varying variables

Note: Variables with a p-value of ≤0.20 (indicated in italics) in the respective bivariate analyses were included in the multivariable analysis. Then, non-significant variables were removed until all variables in the model were significantly associated with the outcome of interest with a p-value of <0.05 as seen above.

All cases of incident syphilis were TPPA confirmed.

In the multivariable analysis after backward selection, older than 24 years of age (adjusted incidence ratio [aIR] 6.18; *P* <0.001), men reporting having sex with a man (aIR 4.63; *P* <0.001), and incident HIV infection (aIR 4.48; *P* = 0.008) were significantly associated with incident syphilis. HIV infection at the baseline visit did not remain significant in the final multivariable model.

Sensitivity analyses were performed to exclude the participants with probable reinfection from both the bivariate and multivariate analyses, and there was no difference in the findings. These models were also done for syphilis reinfection alone, and similar results were found (data not shown).

### Models exploring incident HIV and risk of syphilis incidence

Adjustment for socio-demographic variables for incident HIV decreased the magnitude of effect with an incidence ratio closer to 1.0, but significance of association was maintained (see Table 3). After adjustment for sexual risk behaviors without backward selection, which included variables such as compensated sex, number of sex partners, any unprotected sex, not being in a stable partnership and age of sexual debut, incident HIV no longer was associated with incident syphilis.

**Table 3:** Relationship of HIV incidence and incident syphilis infection among high-risk men in Peru.

		HIV positive at baseline		HIV incidence	
	HIV negative	Cumulative Incidence Ratio (95% CI)	p value	Cumulative Incidence Ratio (95% CI)	p value
Crude	ref	3.77 (2.04–6.96)	<0.001	**5.84 (1.74–19.61)**	**0.004**
Adjusted for participant type, age*	ref	1.35 (0.73–2.52)	0.343	**4.48 (1.47–13.60)**	**0.008**
Adjusted for participant type, age, SES	ref	1.37 (0.73–2.55)	0.325	**4.38 (1.40–13.68)**	**0.011**
Adjusted for participant type, age, SES, sexual risk behaviors	ref	1.07 (0.56–1.06)	0.282	2.27 (0.55–9.26)	0.255
Adjusted for participant type, age, SES, sexual risk behaviors, substance use	ref	1.28 (0.66–2.48)	0.464	2.77 (067–11.46)	0.160

* Same model as the backward selection model shown in Table 2

SES: high school education, stable work, food instability

Sexual risk behaviors: Compensated sex, no. of sex partners, any unprotected sex, stable partnership, age of sexual debut.

## Discussion

We report a high rate of syphilis reinfection among high-risk men who have evidence of previous syphilis infection. Behavioral factors such as male same sex behavior and higher number of sex partners were associated with incident syphilis. Additionally, our findings highlight the close relationship between incident syphilis and incident HIV.

Syphilis infection remains an urgent public health problem, despite widely available and low-cost diagnostics and treatment options. In our cohort, syphilis reinfection accounted for three quarters of incident syphilis cases as indicated by baseline reactive titers and appropriate increase of titers reflective of new infection. This supports the idea that there is a higher risk core group of “repeaters” who are reinfected with syphilis [11, 18]. This calls for focused prevention of syphilis beyond periodic testing among high-risk individuals. Innovative strategies including effective partner notification using social media, testing, treatment, promotion of safe sex practices and risk minimization are needed to reach this core group [19–24].

Though sexual risk behaviors did not remain significant in the final models, these risk behaviors did confound the association between the main finding of incident HIV and incident syphilis. This finding has also been reported by the recent longitudinal analysis of high-risk men enrolled in the iPrex trial in multiple sites, including Peru [3]. It is known that there is a synergistic relationship between HIV and syphilis that goes beyond risky behaviors that increases risk of acquisition of both of these STIs [25]. This relationship is not fully understood and various biological hypotheses exist, such as disruption of mucosal barriers through ulcer formation and recruitment of HIV susceptible inflammatory cells to the genital tract [6, 7, 26]. The effective prevention of syphilis and HIV infection are ultimately interrelated. Understanding the factors associated with syphilis infection will help target and improve prevention efforts. Specific counseling to avoid co-infection should be emphasized to all high-risk individuals. Additionally, efforts to combine HIV and syphilis prevention interventions, such as dual diagnostic testing, have been suggested as part of pregnancy screening [27, 28].

There are several limitations to this analysis. Firstly, our definition of syphilis infection was solely based on RPR interpretation. Some participants who remain uninfected may be misclassified as reinfections. However, strict criteria for syphilis reinfection was used consistently to all participants to minimize this potential error and as previously mentioned, separate analyses were performed excluding those with probable reinfection with similar findings. Secondly, by the nature of the definition of syphilis reinfection, if a previous infection that occurred before the study began was successfully treated, we would not have captured potential reinfections, which further decreases our power to detect differences. We attempted to address this limitation by including the parallel analysis of incident syphilis, as risk factors for incident syphilis are likely to be similar to those for syphilis reinfection. Thirdly, this longitudinal analysis did not account for the exact time when the syphilis reinfection occurred. All infections were considered at annual follow-up visits, even if the rise in RPR occurred during the interim visits between the follow-up visits. However, most syphilis reinfection occurred at annual visits (94%). This is most likely due to the fact that the definition of syphilis reinfection requires time for RPR titer decrease status post treatment, so interim visits which occurred at four month intervals may not provide enough time for RPR titer changes. Also, participants with non-reactive RPR titers were not given interim visits, so new seropositivity would only be observed at annual visits. This differential follow-up of syphilis cases is also another limitation of this secondary analysis.

Syphilis continues to be an uncontrolled public health problem with high rates of syphilis reinfection among high-risk groups in Peru. Considering its serious sequelae and role of increasing HIV transmission, controlling syphilis should be emphasized with urgency. HIV and syphilis prevention efforts should be coordinated and further studied to elucidate the behavioral and biological components between the two infections are necessary.
